# Accidents in Children Under 5 Years in Isfahan, Iran

**Published:** 2014-06

**Authors:** Rohollah Lak, Abolfazl Hajari, Mohsen Naderi Beni

**Affiliations:** 1 Health Technology Assessment, Tehran University of Medical Sciences, Tehran; 2 Center of Disease Control, Tiran & Karvan Center of Health; 3 Center of Disease Control, Chadegan Center of Health, Isfahan University of Medical Sciences, Isfahan, Iran.

**Keywords:** Accident; Under 5 years; Isfahan, Iran

Dear Editor,

Accidents and incidents are mainly the third cause of death in all ages and are so first cause of death in ages under 40 years in the entire world, while, these are second cause of death after coronary and heart diseases in Iran^[1]^. Accidents are responsible for the annual death of more than 10,000 people on an international scale and the cause of approximately 10 percent of all children’s admissions to hospitals^[2]^. 

 In this cross sectional study we studied 33204 patients referred to the health and treatment centers, emergency rooms and hospitals of Isfahan Medical University of Sciences, between 2006 and 2010; and analyzed collected data by χ^2^ test in SPSS-16 software.

 From 33204 children under 5 years, 61.8% were males and 38.2% females, 12.67% were under 1 year and 87.33% 1-5 years old. 

 Impact was the most prevalent of the accident types (Table 1). The most frequent places in which accidents occurred were: in the house (66.37%), and on the streets (21.25%) and they occurred least frequently in working places (0.59%). Drowning had the highest death rate (35.48 %) followed by automobile accident (1.12%).

 This study indicates that different variables are responsible in accident occurrence and the relationship between variables such as gender, age, location and the result of the accident with the accident type is quite obvious. As mentioned before, the relationship between some of the variables are shown from a statistical viewpoint. Boys had approximately twice as many accidents as girls.

 Also accident occurrence was much higher in urban than in rural areas, may be because of 

higher population in the city. The largest amount of accidents resulted in recovering of the patients after treatment, a small number (almost 0.03%) were disabled and 0.27% died.

 Each year averagely 6640 accidents involve children less than 5 years of age which mostly could be prevented. To prevent accidents it is necessary to constantly educate individuals, families, schools and society to avoid encountering dangers related to injuries. Also it is essential to engage in aiding and emergencies, to teach first aids and basics in all levels in accidents which have more risk of death.

**Table 1:** Accident types in children less than 5 years old

**Accident**	**< 1 year**	**1-5 years**
	**n (%)**	**n (%)**
**Motorcycle rder**	136 (0.41)	1432 (4.31)
**Automobile driver**	280 (0.84)	1592 (4.79)
**Impact**	1383 (4.17)	11630 (35.03)
**Falling from heights**	1102 (3.32)	7176 (21.61)
**Violence**	9 (0.03)	121 (0.36)
**Burning**	627 (1.89)	1445 (4.35)
**Pedestrian **	101 (0.30)	1863 (5.61)
**Snake and scorpion bite**	10 (0.03)	53 (0.16)
**Animal bite**	6 (0.02)	214 (0.64)
**Poisoning**	232 (0.70)	978 (2.95)
**Suicide**	4 (0.01)	24 (0.07)
**Electrocution**	9 (0.03)	46 (0.14)
**Drowning**	2 (0.01)	29 (0.09)
**Other**	306 (0.92)	2394 (7.21)
**Total**	4207 (12.67)	28997(87.33)
